# Inhibitory Effect of *Acer truncatum* Bunge Seed Coat Extract on Fatty Acid Synthase, Differentiation and Lipid Accumulation in 3T3-L1 Adipocytes

**DOI:** 10.3390/molecules27041324

**Published:** 2022-02-16

**Authors:** Yan Liang, Fan Kong, Xiaofeng Ma, Qingyan Shu

**Affiliations:** 1 Key Laboratory of Plant Resources and Beijing Botanical Garden, Institute of Botany, Chinese Academy of Sciences, Beijing 100093, China; yanliang@cupes.edu.cn (Y.L.); kongfan20@mails.ucas.ac.cn (F.K.); 2 School of Kinesiology and Health, Capital University of Physical Education and Sports, No. 11 Beisanhuanxi Road, Beijing 100191, China; 3 College of Life Sciences, University of Chinese Academy of Sciences, No. 19A Yuquan Road, Beijing 100049, China

**Keywords:** fatty acid synthase, *Acer truncatum* Bunge, inhibitor, obesity

## Abstract

*Acer truncatum* Bunge is now widely cultivated throughout the world. Fatty acid synthase (FAS) is a potential target in the treatment of both obesity and cancer. Only a few FAS inhibitors have been reported. In this study, the inhibitory effect of *A. truncatum* seed coat (ESA) on FAS and the inhibition mechanisms were investigated using a FAS activity assay and an enzyme kinetics study. The main chemicals of ESA were analyzed with UPLC-MS/MS. The effects of ESA on 3T3-L1 adipocyte differentiation and lipid accumulation were investigated using Oil red O staining. We first identified seven main compounds (quinic acid, malic acid, gentisic acid, procyanidin dimer, procyanidin trimer, catechin, and quercetin) from 50% ethanol extracts of seed coats of *A. truncatum* (ESAs), which were then found to inhibit 3T3-L1 adipocyte differentiation at the concentration of 50 μg/mL. ESA obviously reduced the visible triglyceride droplets accumulation, and dramatically decreased the number of the adipocytes at a comparatively high concentration. It is suggested that the effects are due to the inhibition of FAS by ESA; FAS activity is inhibited by ESA at a half inhibition concentration (IC_50_) of 0.57 μg/mL, which is lower than that of classically known FAS inhibitors. Meanwhile, ESA displayed different inhibition kinetics and reacting sites for FAS. These results provide new clues for the development of novel products for obesity treatment and a scientific basis for the full use of byproducts for future industrial production of vegetable oil.

## 1. Introduction

Obesity is prevailing at an alarming rate in not only the developed countries but the developing countries. Many diseases are considered to be related to obesity, such as cardiovascular disease [1], type 2 diabetes [2] and cancer [3]. Thus, novel treatments for obesity are valuable to reduce obesity-related health problems.

Adipose tissue is often considered the core of obesity. Increase in adipose tissue mass is related to both the size and number of adipocytes (hypertrophy and hyperplasia), which all rely on the differentiation from preadipocytes into adipocytes. In humans, it has been reported that FAS gene expression in adipocytes is associated with obesity and diabetes [4]. Similarly, typical inhibitors of FAS, such as C75 and cerulenin, block the differentiation of 3T3-L1 cells and prevent obesity [5]. This progress suggests a new way to treat obesity by targeting FAS in adipocytes.

Animal FAS, which is mainly expressed in liver and adipose tissue, de novo synthesizes fatty acid from acetyl-CoA and malonyl-CoA in the presence of NADPH [6]. This enzyme is recently recognized as a potential therapeutic target for obesity and certain kinds of cancer [7,8]. It was reported that treatment with FAS inhibitors reduced the food intake and body weight of obese mice [7]. Some natural compounds or extracts from plants showed a potent inhibitory effect on FAS and anti-obesity activity [9]. Therefore, more natural inhibitors of FAS can be expected for the treatment of obesity.

*Acer truncatum* Bunge, which belongs to the genus *Acer* of the family Aceraceae, is planted widely in the world. Traditionally, it has been used by different language groups such as Mongolian, Tibetan and Korean to prevent cardiovascular and cerebrovascular diseases and to treat skin trauma [10]. *A. truncatum* has been used as a traditional herbal medicine in northern China for centuries [11]. Most of the pharmacological activities of *A. truncatum* have been reported, showing its potential in the development of new drugs or health products [12]. However, detailed information on the molecular mechanism, metabolic activity and toxicology of active ingredients is limited. Besides being used as traditional medicine, *A. truncatum* has important edible value. Its leaves have been used as medicinal herbs and as the raw material for maple tea in China, and there is a tradition of directly eating its fried seeds [13]. In 2011, its seed oil was authenticated as a new food resource by the Ministry of Health of China. Its seed coat is a byproduct that is generally abandoned as waste during seed oil production. Previous studies focused on chemical constituent detection and isolation with various bioactivities, such as nervonic acid, flavonoids, flavonoid glycosides, phenylpropanoids and tannins [14,15,16,17]. About 16 main fatty acid components were detected in *A. truncatum* oil, and the contents of these substances varied greatly. The content of linoleic acid is the highest, accounting for about 35% of the total fatty acids, and the content of nerve acid is as high as 5–6% [15]. Some other bioactivities have been neglected, such as those related to the treatment of human chronic disease. 

In this work, we report that the extracts of seed coat of *A. truncatum* (ESA) effectively inhibit differentiation of 3T3-L1 adipocytes by reducing the size and number of these cells. The investigation showed that ESA inhibits FAS activity significantly. The results show the potential of ESA as a valuable resource to treat obesity and provide a strategy for making full use of byproducts, since their biomass accounts for 30% that of seeds.

## 2. Results

### 2.1. Identification of ESA through UPLC-QTOF-MS

**Figure 1:**
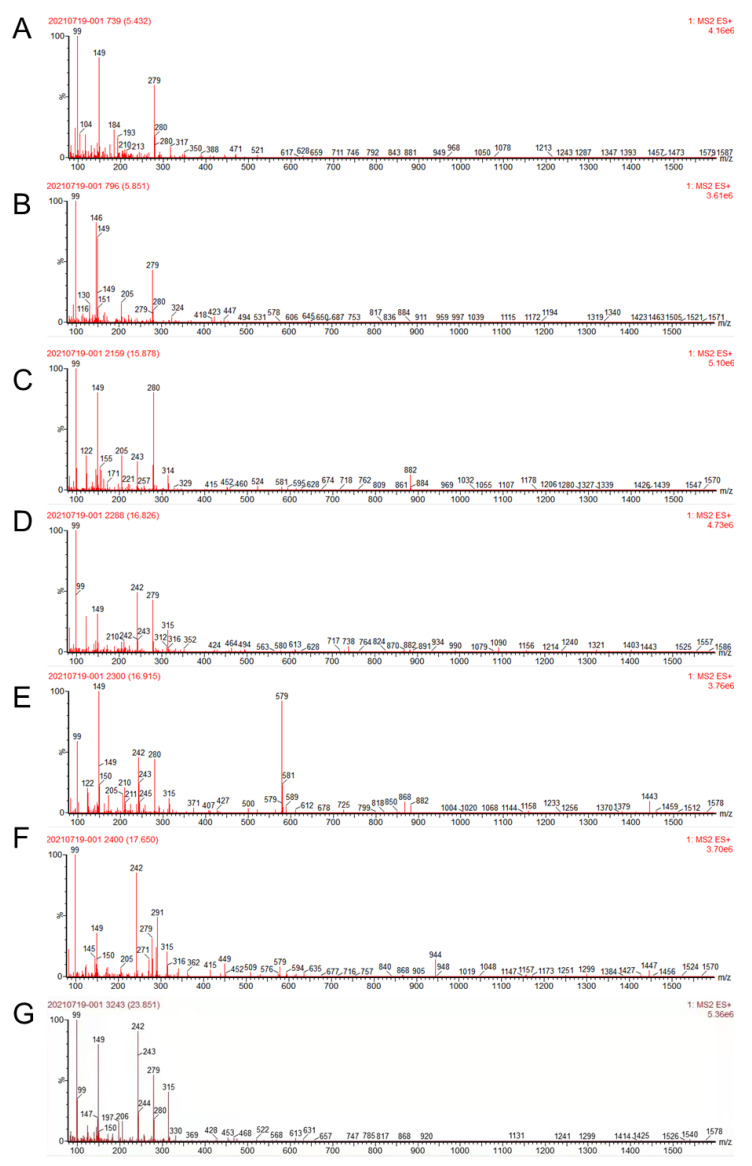
The MS spectra of compounds detected in ESA. (**A**) The MS spectrum of quinic acid; (**B**) The MS spectrum of malic acid; (**C**) The MS spectrum of gentisic acid; (**D**) The MS spectrum of procyanidin dimer; (**E**) The MS spectrum of procyanidin dimer; (**F**) The MS spectrum of catechin; (**G**) The MS spectrum of quercetin.

**Figure 2:**
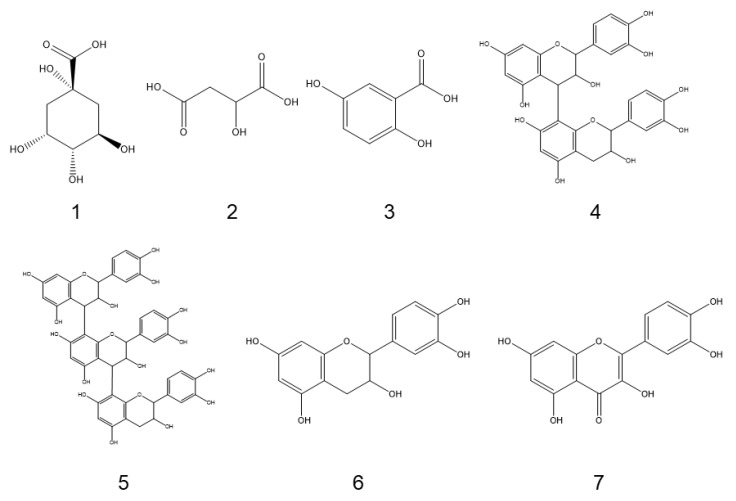
Chemical structures of compounds identified in ESA.

Based on absorption from 200–800 nm, the compounds’ absorption was mainly detected in the initial 20 min; seven obvious absorption signals were obtained. Based on a reference and Mass spectrum, we identified seven chemicals (Table 1; Figure 1), among which compounds 1 (t_R_ = 5.436 min), 2 (t_R_ = 5.855 min), and 3 (t_R_ = 15.874 min) showed ([M−H]^−^) at *m*/*z* 191, 133 and 153 and were identified as quinic acid, malic acid, and gentisic acid, respectively. Compound 4 (t_R_ = 16.823 min) and 5 (t_R_ = 16.911 min) demonstrated ([M−H]^−^) at *m*/*z* 577 and 865 were identified as procyanidin derivates; compound 6 (t_R_ = 17.654 min) and 7 (t_R_ = 23.855 min) with ([M−H]^−^) at *m*/*z* 289 or 301 were identified as catechin and quercetin, respectively. The chemical structures are shown in Figure 2. Some compounds were not identified due to less content.

### 2.2. The Inhibitory Effects of ESA on Adipocyte Differentiation

**Figure 3:**
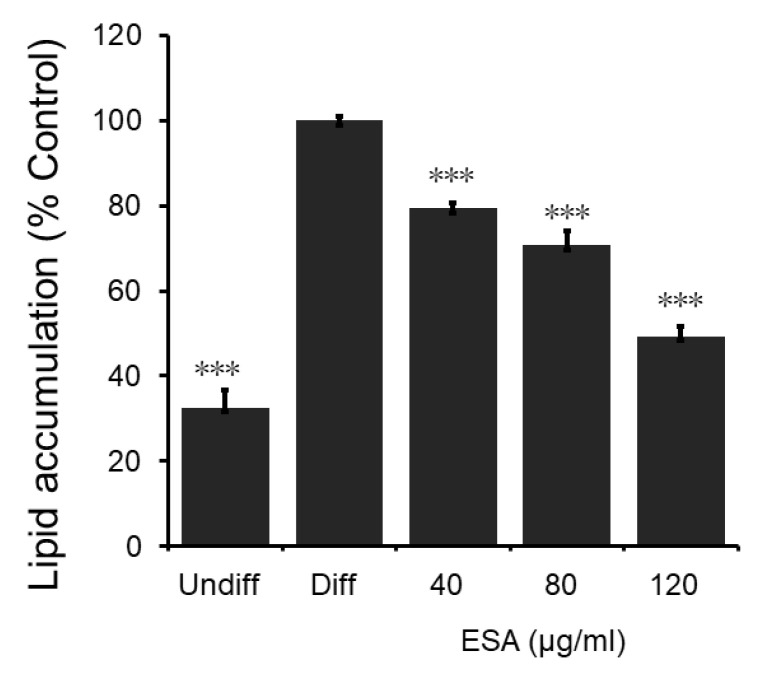
Inhibitory effects of ESA on lipid accumulation. Stained oil droplets in the cell were dissolved in isopropanol, and spectrophotometrically measured at an absorbance of 520 nm. ESA inhibits 3T3-L1 intracellular triglyceride accumulation in a dose-dependent manner; *n* = 4, mean ± SD; Undiff: undifferentiated cells; Diff: differentiated cells; the *p* value obtained using a 2-tailed T-test. *** = *p* < 0.001 compared to Diff.

The results of oil red O staining show that the intracellular triglyceride content is obviously reduced by ESA (Figure 3). The 40, 80, and 120 μg/mL of ESA inhibited the cell lipid accumulation to 79.7%, 71.8% and 50.9% of that of the controlling differentiated adipocyte, respectively. They demonstrate that the inhibition of 3T3-L1 cell differentiation by ESA is in a dose-dependent manner.

**Figure 4:**
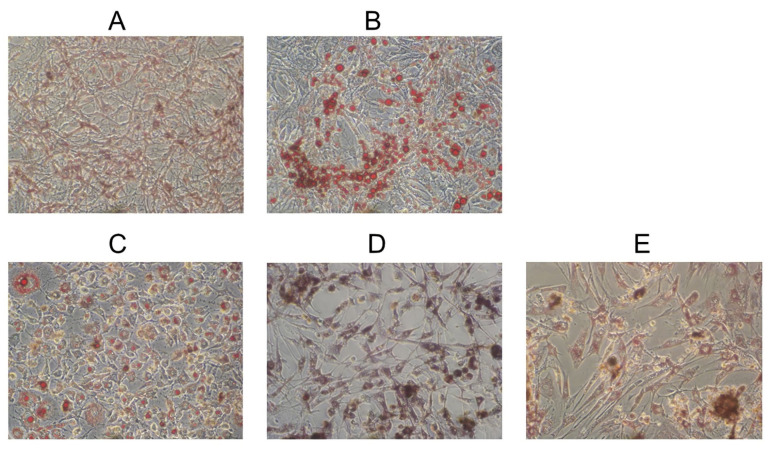
Effects of ESA during differentiation on morphology of 3T3-L1 cells. Undifferentiated cells are shown in (**A**) and differentiated cells in (**B**). Cells differentiated in the presence of 40 μg/mL ESA (**C**), 80 μg/mL ESA (**D**), and 120 μg/mL ESA (**E**). Photos were taken after the oil red O staining, and original magnification was 100×.

As shown in Figure 4, undifferentiated preadipocytes (Figure 4A), fibroblasts, and no lipid droplets were visible under microscopy. With the differentiation of 3T3-L1 cells, multiple visible lipid droplets were developed in the cell which became the differentiated adipocytes (Figure 4B). ESA at 40 μg/mL (Figure 4C) inhibited the adipocyte differentiation obviously since fewer lipid droplets were observed in the cell. ESA at 80 μg/mL (Figure 4D) and 120 μg/mL (Figure 4E) had an even greater effect on the differentiation of adipocytes. From the photograph of adipocytes after oil red O staining, not only was the differentiation greatly inhibited and the triglyceride droplets disappeared, but also the number of adipocytes decreased in the presence of high concentrations of ESA.

### 2.3. Inhibition on Overall Reaction and β-Ketoacyl Reduction of FAS by ESA

**Figure 5:**
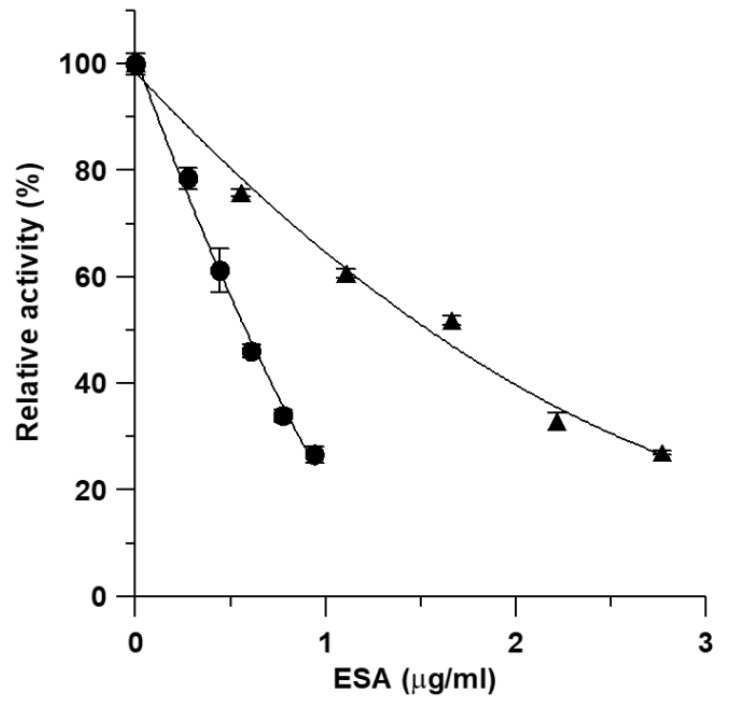
Effects of ESA on FAS activity. The inhibition of FAS was measured in the presence of various concentrations of the inhibitor: (●) inhibition for the overall reaction; (▲) inhibition for the *β*-ketoacyl reduction. Data are the means from three experiments.

Inhibitory activity of ESA on the overall reaction and *β*-ketoacyl reduction of FAS was assayed in vitro. Figure 5 shows the inhibition of the overall reaction and *β*-ketoacyl reduction by various concentrations of ESA. The IC_50_ values from the plots were 0.57 and 1.71 μg/mL for the overall reaction and *β*-ketoacyl reduction, respectively.

### 2.4. Inhibition Kinetics Studies

**Figure 6:**
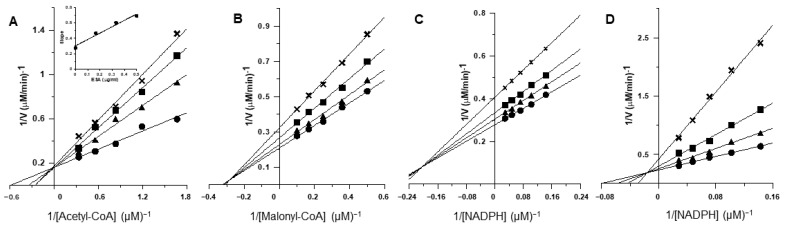
Lineweaver-Burk plot for inhibition of FAS by ESA: the overall reaction of FAS was measured. (**A**) Concentrations of malonyl-CoA and NADPH were fixed at 10 and 35 μM, respectively. Acetyl-CoA was the variable substrate. The concentrations of ESA were: 0 μg/mL (●), 0.17 μg/mL (▲), 0.33 μg/mL (■), and 0.50 μg/mL (×). (**B**) Concentrations of acetyl-CoA and NADPH were fixed at 3 and 35 μM, respectively. Malonyl-CoA was the variable substrate. The concentrations of ESC were: 0 μg/mL (●), 0.11 μg/mL (▲), 0.22 μg/mL (■), and 0.33 μg/mL (×). (**C**) Concentrations of acetyl-CoA and malonyl-CoA were fixed at 3 and 10 μM, respectively. NADPH was the variable substrate, and the concentrations of ESA were the same as in (**B**). (**D**) Lineweaver-Burk plot for inhibition of the reaction using ethyl acetoacetate as substrate by ESA. Concentrations of ethyl acetoacetate were fixed at 40 mM, and NADPH was the variable substrate. The concentrations of ESA were: 0 μg/mL (●), 0.55 μg/mL (▲), 1.11 μg/mL (■), and 1.66 μg/mL (×).

To elucidate the mechanism of inhibition on the overall reaction of FAS by ESA, the inhibition kinetics were investigated. Lineweaver-Burk plots for inhibition of FAS by ESA were shown in Figure 6. Three families of straight lines were yielded for the substrates of FAS. The dissociation constants, Ki, were obtained from the secondary plot of slopes of these lines versus ESA concentrations. The dissociation constant for the inhibitor-enzyme-substrate complex, Ki’, was calculated from the secondary plot of *Y*-axis intercepts versus ESA concentration. In Figure 6A, acetyl-CoA was the variable substrate, and the lines intersected on the *Y*-axis. The result indicates that ESA inhibited FAS competitively with acetyl-CoA. The type of ESA approached a typical noncompetitive substrate such as malonyl-CoA, for lines have a point intersection nearly on the *X*-axis (Figure 6B). The lines for NADPH (Figure 6C) have the point intersection in the second quadrant, indicating the inhibition had a mixed competitive and noncompetitive effect. 

The inhibition kinetics of the *β*-ketoacyl reduction of FAS by ESA were also studied. As shown in Figure 6D, lines for NADPH had the point intersection in the second quadrant, indicating the inhibition was of the mixed type of competitive and noncompetitive effects. The inhibitory types of ESA and the dissociation constants of ESA are summarized in Table 2.

## 3. Discussion

In the present work, the potent inhibitory effects of ESA on both adipocytes and FAS were investigated. We first analyzed the constituents of ESA: seven main compounds were identified from ESA by UPLC-MS/MS. It has been demonstrated that ethanol is more effective for antioxidant extraction with higher activity than acetone and water; among extracts, we identified a fewer number of compounds than was reported before [18]. The possible reason may be due to the extraction concentration of methanol or the various seed coat origins. We also identified gentisic acid, which was reported for the first time in ESA [18]. Until now, more studies focused on seed coat components and content as well as antioxidant activity in *Acer* species [18,19,20]. A few studies were on cytotoxic and inhibitory activities on human tumor cell lines, with 15 identified chemicals extracted from dry seeds [21], which was different from this study. In this study, ESA effectively inhibited the 3T3-L1 adipocyte differentiation. Meanwhile, it obviously reduced the size of individual adipocytes caused by the accumulation of triglycerides in the process of development from preadipocytes into adipocytes. Furthermore, we demonstrated that ESA decreased the number of the cells at a comparatively high concentration. We also found that ESA had high inhibitory activity on FAS activity, with an IC_50_ value of 0.57 μg/mL, which is lower than that of EGCG and cerulenin [5]. In addition, although quinic acid was detected in both leaves [22] and seed coats, the inhibitory effect of ESA on FAS activity was stronger than that of the crude extract of *A. truncatum* leaves [22], which indicates that ESA may contain different ingredients, especially active components. The results of UPLC-MS/MS analysis confirm the conclusion.

The inhibition kinetics study of ESA further demonstrated that ESA contains different FAS inhibitors from extracts of the leaves of *A. truncatum* (ELA) [22]. ESA inhibited FAS competitively with acetyl-CoA, and noncompetitively with malonyl-CoA and NADPH. For ELA, the inhibition was noncompetitive with acetyl-CoA, competitive with malonyl-CoA and uncompetitive with NADPH, respectively [23]. In addition, ESA strongly inhibited *β*-ketoacyl reduction of FAS with an IC_50_ value of 1.7 mg/mL; on the contrary, relevant IC_50_ values for ELA were reported as high as 16 mg/mL [22]. These results show that the *β*-ketoacyl reductase domain on FAS is one of the main reacted sites for ESA, but is not for ELA. An HPLC study on ELA showed that the main components of ELA were two flavonoids, which were reported to be FAS inhibitors [24]. Although we have not successfully separated and identified compounds with high inhibitory activity from ESA, it is certain that the inhibitors of FAS in the two extracts are different, despite the similar degree of inhibition on FAS’s overall reaction and 3T3-L1 cells by ESA and ELA [23].

ESA and ELA provide two separate examples of FAS inhibitors reducing 3T3-L1 adipocyte differentiation and accumulation of lipids. Schmid reported that C75, a typical FAS inhibitor, inhibits adipocyte differentiation so as to completely avoid lipid accumulation; in that instance, inhibition of FAS can prevent preadipocyte differentiation and triglyceride accumulation [5]. Therefore, it is suggested that the inhibition of ESA on the 3T3-L1 adipocyte differentiation may due to the inhibition of FAS activity.

Though the particular mechanisms of their effects on adipocytes are still unclear, the extracts from natural plants are safe to use. Both the seeds and leaves of *A. truncatum* have high nutritional and medical value. Its leaves have been used as herbal medicine and some kinds of tea for the treatment of coronary artery cirrhosis, angina pectoris and cerebrovascular diseases in China [25,26,27]. Its seeds are used for extraction of edible oil. The present results prefigure that the extracts of *A. truncatum* seeds may have wide application prospects and be suitable for developing health care products that could make full use of valuable resources.

## 4. Materials and Methods

### 4.1. Reagents

Dulbecco’s modified Eagle’s medium (DMEM) and fetal bovine serum (FBS) were purchased from Gibco BRL. 3-isobutyl-1-methylxanthine (IBMX), insulin, dexamethasone, oil red O, acetyl-CoA, malonyl-CoA, and NADPH were purchased from Sigma (St. Louis, MO, USA). Acetonitrile (≥99.9% purity) used for high performance liquid chromatography (HPLC) analysis was of chromatographic grade (Sigma, St. Louis, MO, USA). All other reagents were local products (Beijing Chemical Reagent Company, Beijing, China) with purity of analytical grade.

### 4.2. Preparation of the Extracts

Seeds of *Acer truncatum* Bunge were collected in the environs of Beijing, China. Fresh seeds were picked up and air dried in the shade. The dry seed coats were smashed to rough powder weighing up to 3 g and in turn extracted 3 times with ultrasound for 20 min with 20 mL 50% ethanol at 25 °C. (As we found that the component inhibiting FAS in the seed coats has a large polarity, 50% ethanol was decided as the best solvent for extraction). Then, the extract was gathered and evaporated under reduced pressure to remove ethanol and yield a brown residue which was further dried in air. All the stock samples were divided and stored at 4 °C.

### 4.3. Identification of Compound of ESA Using UPLC- MS/MS

Approximately 100 µg of ESA was dissolved in 1 mL of methanol and subjected to filtration with a 0.22 µm filter; then 10 µL of the solution was uploaded for further analysis. An Acquity UPLC system (Waters Corporation, Milford, MA, USA) with a Diamonsil C18 column (5 μm, 4.6 mm × 250 mm, Dikma Technologies Inc., Lake Forest, CA, USA) was used for chromatographic separation. The mobile phases A and B used a mixture of acetonitrile and water with 0.05% formic acid, respectively. The gradient elution program was set as follows: 10% A for 5 min at 23.1 °C; 10–100% A for 30 min, 100% A for 5 min; 100–10% A for 5 min. The flow rate was set at 0.5 mL/min. The absorbance measured was 205–800 nm. A Xevo TQ Mass Spectrometer (Waters) with an ESI interface operating in negative ion resolution mode was connected with the UPLC system and a capillary voltage of 2.5 kV was used. The gas flow rate was set at 11.6 L/min, the temperature at 240 °C, and a mass range of 50–1600 *m*/*z* was selected. 

### 4.4. Cell Line and Cultures

Mouse 3T3-L1 preadipocytes were purchased from the Type Culture Collection of the Chinese Academy of Sciences, Shanghai, China. Cells were cultured in DMEM supplemented with 10% fetal bovine serum at 37 °C in the presence of 5% CO_2_. The medium was changed every 2 days. 3T3-L1 preadipocytes were seeded in a 24-well plate and grown for 2–4 days for differentiation. Two days after reaching confluence, the medium was changed to DMEM containing 10% FBS supplemented with 0.5 mM 3-isobutyl-1-methylxanthine, 1 mM dexamethasone, and 1.7 mM insulin (day 0). The cells were treated for 2 days (day 2), and then were cultured in DMEM containing 10% FBS and 1.7 mM insulin for another 2 days. Thereafter (day 4), the cells were cultured in DMEM containing 10% fetal bovine serum to day 8, and the medium was changed every 2 days. The ESA was added at the beginning of the differentiation process and fresh inhibitor was added whenever a medium change was performed.

### 4.5. Oil Red O Staining

Cell differentiation and intracellular lipid accumulation were determined by oil red O staining at day 8 after adipocyte differentiation. The cells were washed twice with phosphate-buffered saline (PBS), and stained with 0.3% (*w*/*v*) oil red O solution in 60% (*v*/*v*) isopropanol for 1 h. After staining, the cells were washed three times with water to remove excess stains. Stained oil droplets in the cell were dissolved in isopropanol and spectrophotometrically measured at an absorbance of 520 nm.

### 4.6. Preparation of FAS and Substrates

Chicken FAS was used. The preparation, storage and use of FAS were performed as described previously [22]. The final purified enzyme was homogeneous by polyacrylamide gel electrophoresis in the presence or absence of SDS. The enzyme and substrate concentrations were determined by absorption measurements using the extinction coefficients according to the method previously described [28]. 

### 4.7. Assay of FAS Activity

The overall reaction and *β*-ketoacyl reduction (KR) of FAS were determined with an Amersham Pharmacia Ultrospec 4300 pro UV-Vis spectrophotometer (Amersham, UK) at 37 °C by following the decrease of NADPH at 340 nm. The reaction mixture used for these reactions has been described previously [28]. Briefly, the assay solution for the overall reaction contained 100 mM potassium phosphate buffer, pH 7.0, 1 mM EDTA, 1 mM dithiolthreitol, 3 mM acetyl-CoA, 10 mM malonyl-CoA, 35 mM NADPH, and 10 mg of FAS in a total volume of 2.0 mL. The *β*-ketoacyl reduction reaction mixture (2 mL) contained 40 mM ethyl acetoacetate, 35 mM NADPH, 1 mM EDTA, 1 mM dithiolthreitol and 10 mg of FAS in 10 mM phosphate buffer at pH 7.0.

### 4.8. Inhibition Studies of the Extracts

The inhibition effect of the extracts was investigated by adding sample solution (1–10 mL) to the reaction system, followed by the addition of FAS solution to initiate the reaction in a total volume of 2 mL. The extracts were dissolved in dimethyl sulfoxide (DMSO) and added to the reaction mixtures described above. The final concentration of DMSO was under 0.5% (*v*/*v*), to avoid the interference with FAS activity. The control activity of FAS with solvent same as the sample only was assayed as A0, the activity added sample was assayed as Ai, and the Ai/A0 was the residual activity. This inhibition was usually reversible and caused by the non-covalent fast combination of inhibitor with enzymes. The extent of inhibition by the addition of inhibitors was measured by reference to the half inhibition concentration (IC_50_). The IC_50_ was obtained from a plot of residual activity versus inhibitor concentration.

### 4.9. Enzyme Kinetics Study

Possible interference by inhibitors at each substrate-binding site was examined by holding the concentration of the inhibitor at a constant value, and the effect of increasing one substrate concentration (with the other substrate concentrations fixed) on the initial reaction rate was measured.

### 4.10. Statistics

Data were expressed as means ± standard deviations (SD). The unpaired Student’s *t* test was used to compare the means of two groups. *p* values of 0.05 or less were considered to be statistically significant.

## 5. Conclusions

In conclusion, ESA is a novel FAS inhibitor. ESA inhibits preadipocyte proliferation and reduces lipid accumulation. Meanwhile, ESA displays different inhibition kinetics and reacting sites on FAS compared to known classic FAS inhibitors. These results provide new clues for the development of novel products for obesity treatment and a scientific basis for the full use of byproducts for future industrial production of vegetable oil.

## Figures and Tables

**Table 1:** Identification of compounds of ESA using UPLC-MS/MS.

Number	t_R_ (min)	Compound	Molecular Formula	[M−H]^−^*m*/*z*	MS/MS Characteristic Fragments	References
1	5.436	Quinic acid	C_7_H_12_O_6_	191	85	[18]
2	5.855	Malic acid	C_4_H_6_O_5_	133	115	[18]
3	15.874	Gentisic acid	C_7_H_6_O_4_	153	109	[18]
4	16.823	Procyanidin dimer	C_30_H_26_O_12_	577	407/289	[18]
5	16.911	Procyanidin trimer	C_45_H_38_O_18_	865	289/577	[18]
6	17.654	Catechin	C_15_H_14_O_6_	289		[18]
7	23.855	Quercetin	C_15_H_10_O_7_	301	151	[18]

**Table 2:** Inhibition types for ESA against every substrate of FAS.

Substrates	ESA		
	Type	K_i_ (μg/mL)	K_i_’ (μg/mL)
Acetyl-CoA	Competitive	0.36	-
Malonyl-CoA	Noncompetitive	0.49	0.58
NADPH	Mixed competitive and noncompetitive	0.50	0.71
NADPH (*β*-ketoacyl reduction)	Mixed competitive and noncompetitive	0.23	1.95

## Sample Availability

Samples of the compounds 1–7 are not available from the authors.
